# Multiple Sessions of Transcranial Direct Current Stimulation (tDCS) Reduced Craving and Relapses for Alcohol Use: A Randomized Placebo-Controlled Trial in Alcohol Use Disorder

**DOI:** 10.3389/fphar.2018.00716

**Published:** 2018-07-03

**Authors:** Jaisa Klauss, Quézia S. Anders, Luna V. Felippe, Michael A. Nitsche, Ester M. Nakamura-Palacios

**Affiliations:** ^1^Program of Post-Graduation in Physiological Sciences, Laboratory of Cognitive Sciences and Neuropsychopharmacology, Health Sciences Center, Federal University of Espírito Santo, Vitória, Brazil; ^2^Leibniz Research Centre for Working Environment and Human Resources, Dortmund, Germany; ^3^Department of Neurology, University Medical Hospital Bergmannsheil, Bochum, Germany

**Keywords:** alcohol dependence, tDCS, dorsolateral prefrontal cortex, craving, relapses

## Abstract

**Background:** Transcranial direct current stimulation (tDCS), a non-invasive brain stimulation technique, has been studied as an adjunctive therapeutic agent for alcohol dependence. In a previous study, we showed that five consecutive sessions of tDCS applied bilaterally over the dorsolateral prefrontal cortex (dlPFC) reduced relapse to the use of alcohol in alcohol use disorder (AUD) outpatients. However, no changes on craving scores were observed. In the present study, we investigated if an extended number of sessions of the same intervention would reduce craving and relapses for alcohol use in AUD inpatients.

**Methods:** Thus, a randomized, double-blind, sham-controlled, clinical trial with parallel arms was conducted (https://clinicaltrials.gov/ct2/show/NCT02091284). AUD patients from two private and one public clinics for treatment of drug dependence were randomly allocated to two groups: real tDCS (5 × 7 cm^2^, 2 mA, for 20 min, cathodal over the left dlPFC, and anodal over the right dlPFC) and sham-tDCS. Real or sham-tDCS was applied once a day, every other day, in a total of 10 sessions. Craving was monitored by a 5-item obsessive compulsive drinking scale once a week (one time before, three times during and once after brain stimulation) over about 5 weeks.

**Results:** Craving scores progressively decreased over five measurements in both groups but were significantly reduced only in the real tDCS group after treatment. Corrected Hedges' within-group (initial and final) effect sizes of craving scores were of 0.3 for the sham-tDCS and of 1.1 for the real tDCS group. Effect size was 3-fold larger in the real tDCS group. In addition, the between-group analysis on craving score difference was nearly significant, and the effect size was 0.58, in favor for a larger effect in the real tDCS group when compared to sham-tDCS. Furthermore, in a 3-months follow-up after intervention, 72.2% of sham-tDCS group relapsed to the alcohol use whereas 72.7% of tDCS group were abstinent.

**Conclusions:** Multiple sessions of bilateral prefrontal tDCS were well tolerated with no significant adverse events. Thus, extended repetitive bilateral tDCS over the dlPFC is a promising adjunctive clinical tool that could be used to reduce alcohol craving and relapses and facilitate alcoholism cessation.

## Introduction

Alcohol is a highly addictive substance and alcohol dependence is a chronically relapsing disorder. It induces tolerance such that increased doses of the alcohol are required to achieve the desired effects and is associated with adverse symptoms during its acute withdrawal. Progressing disease is accompanied with neglect of alternative interests, and social, family, and occupational activities. Attempts to quit are often unsuccessful and the patient continues to use the substance despite knowledge of physical and/or psychological harm caused by alcohol (DSM-5, 2013). Alcohol dependence is thus a debilitating disorder that harms not only the individual, but inflicts significant costs to society, including loss of productivity, security challenges, crime and lawlessness, increasing health care costs, and a myriad of negative social consequences (Daley, 2013).

Craving is a common manifestation in all drug addictions. It is defined as the “pressing, urgent and irrepressible desire to give in to the substance” (Grall-Bronnec and Sauvaget, 2014), resulting in an uncontrolled urge to consume a drug, with strong obsessions about and irresistible compulsions to use (Robinson and Berridge, 1993), even when the individual is well aware of the consequences that its use can bring to his life. Craving is now considered as part of the diagnosis criteria for Substance Use Disorders under the A4 criteria in the DSM-5 (O'brien, 2011; DSM-5, 2013; Lupi et al., 2017). One reason to add craving to the diagnostic criteria was that it activates brain regions related to the reward system (Wilson et al., 2004; Heinz et al., 2009). Indeed, craving can be caused by an alteration of the relevant brain circuitry, that may persist even when the individual is not currently using the substance, but is exposed to stimuli that are associated with it (Volkow et al., 2011; DSM-5, 2013; Koob and Volkow, 2016), constituting a recognized central driving force for successive relapses and perpetuation of drug use (Self, 1998; Weiss, 2005).

Psychosocial and pharmacological approaches, although essential, have shown limitations and modest efficacy in the treatment of alcohol dependence (Miller et al., 2011). Therefore, the development of more effective treatments or alternatives improving the efficacy of the current approaches is highly desired.

Transcranial direct current stimulation (tDCS) is a non-invasive brain stimulation technique in which a weak current is applied to the brain for several minutes through electrodes, resulting in a polarity-dependent modulation of brain activity (Nitsche et al., 2008; Den Uyl et al., 2015). Considering that a single bilateral tDCS, either left cathodal/right anodal or left anodal/right cathodal, over the dorsolateral prefrontal cortex (dlPFC) showed to reduce alcohol craving in AUD patients (Boggio et al., 2008), but the repetitive unilateral anodal tDCS over the left dlPFC increased instance of relapses in AUD patients (da Silva et al., 2013), in the following study in AUD outpatients, we applied five consecutive sessions of bilateral tDCS, having the cathodal electrode over the left and the anodal over the right dlPFC, and showed a reduced probability of relapse to the use of alcohol (Klauss et al., 2014). Half (50%) of the AUD patients treated with tDCS, as compared to only 11.8% of subjects from the sham-tDCS group, were completely abstinent at the end of 6 months following the intervention. However, in this study craving during the period of brain stimulation was not significantly changed (Klauss et al., 2014).

The extension of tDCS sessions may clinically matters as 10 daily sessions have shown to result in more effective and long-lasting effects than 5 daily sessions (Valle et al., 2009; Kuo et al., 2014). Therefore, in the present study we aimed to investigate whether an intensified intervention with ten sessions of tDCS bilaterally applied over the dlPFC would reduce craving for alcohol use in AUD inpatients.

## Materials and methods

We report this clinical trial according to CONSORT guidelines. This trial was registered under Clinical Trials.gov number NCT02091284.

### Participants

AUD patients of both genders were successively recruited between June of 2015 and January of 2018 from three specialized clinics for drug dependence treatment, one public, and two privates, from the State of Espírito Santo, Brazil. These specialized services applied standard protocols for the treatment of drug addiction, consisting of psychosocial approaches–conducted by a professional team of psychologists, nurses, social workers and physicians, and pharmacotherapy, including benzodiazepines, vitamin B, disulfiram and, when necessary, antidepressants, anxiolytics, antihypertensive, and gastric medications, and folic acid. Two dropouts occurred after randomization. One dropout had to be hospitalized due to clinical instability and another missed many stimulation sessions.

The inclusion criteria for this study were: (1) male and female patients over the age of 18 years; (2) met criteria for alcohol dependence according to the Classification of Mental and Behavioral Disorders (ICD-10) and the Diagnostic and Statistical Manual of Mental Disorders, fifth edition (DSM-5), as determined by clinical evaluation; (3) in stable clinical condition with no need for emergency care; (4) able to read, write, and speak Portuguese; and (5) without severe withdrawal signs or symptoms at baseline.

Exclusion criteria included: (1) a condition of intoxication or withdrawal due to a substance other than alcohol, (2) unstable mental or medical disorder or substance abuse or addiction other than alcohol dependence, except nicotine and/or caffeine or history of marijuana use during adolescence; (3) diagnosis of epilepsy, convulsions, or delirium tremens during the abstinence of alcohol; (4) a previous history of drug hypersensitivity or adverse reactions to diazepam or other benzodiazepines and haloperidol; (5) any contraindication for electrical brain stimulation procedures such as electronic or metal implants.

Ethical approval was provided by the Brazilian Institutional Review Board of the Federal University of Espírito Santo (CAAE 19403713.6.0000.5060), Brazil, and it was registered in clinicaltrials.gov (https://clinicaltrials.gov/ct2/show/NCT02091284). The study was conducted in strict adherence to the Declaration of Helsinki and is in accordance with the ethical standards of the Committee on Human Experimentation of the Federal University of Espírito Santo, ES, Brazil, where this study was conducted. Subjects were fully informed about the experimental protocol and voluntarily signed an informed consent form before the start of the study.

### Direct current (DC) stimulation

The intervention in this clinical trial was transcranial DC stimulation (tDCS). In each session, tDCS was applied via two carbonated silicone electrodes (35 cm^2^) with a thick layer of high-conductive EEG paste on the contact surface connected to a DC-Stimulator (DC-Stimulator Plus, NeuroConn, Ilmenau, Germany) for 20 min with fade-in and fade-out periods of 30 s each. Intensity was set to 2 mA.

**Figure 1:**
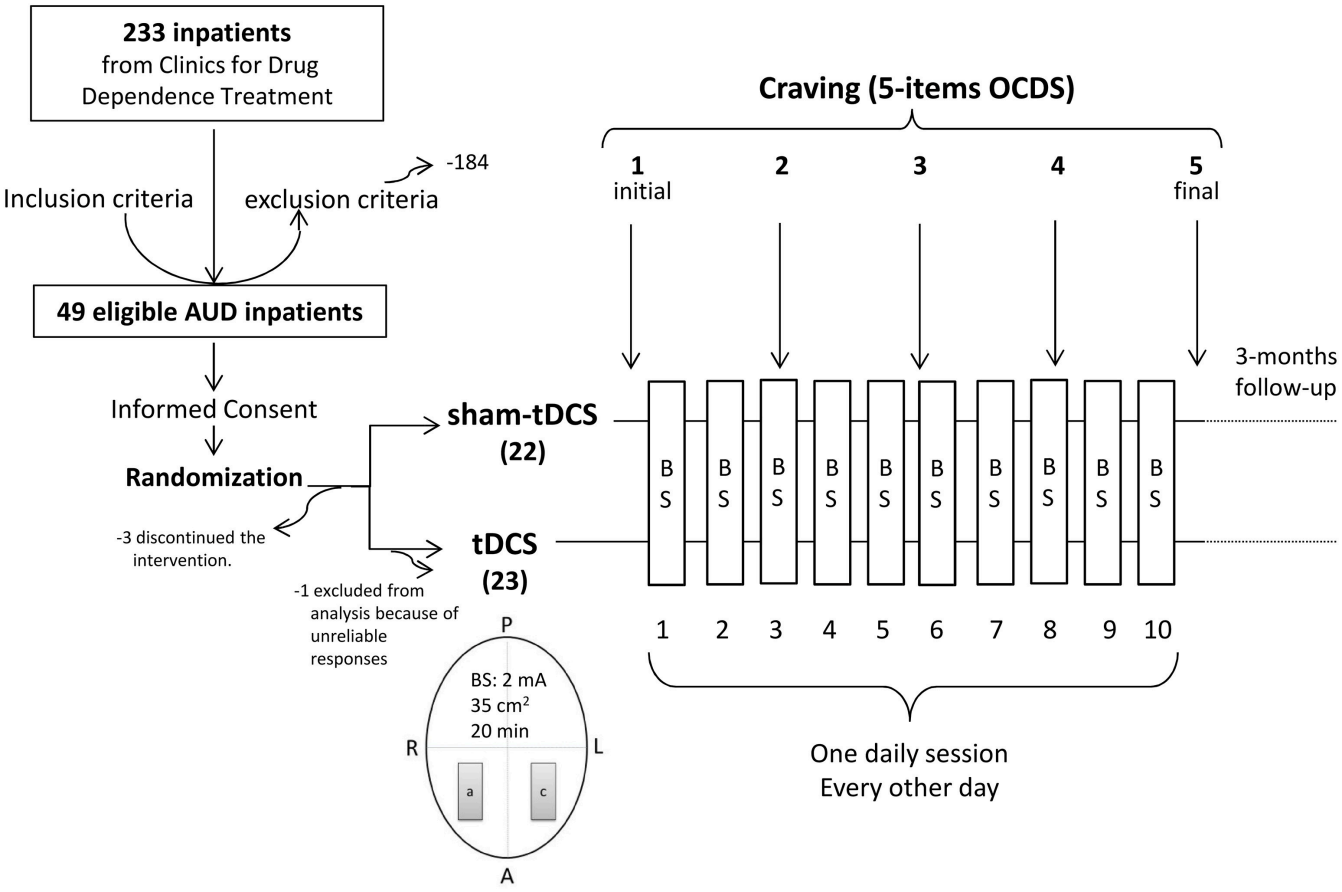
Diagram of the general procedure: eligible Alcohol Use Disorder (AUD) patients were recruited from clinics for treatment of drug dependence, signed the Term of Consent and were randomized to receive repetitive bilateral (cathode left/anode right over the dorsolateral Prefrontal Cortex) transcranial Direct Current Stimulation (tDCS, 2 mA, 35 cm^2^, stimulation for 20 min) or placebo (sham-tDCS) every other day in a total of 10 sessions. Craving to the use of alcohol was examined by 5 items from the Obsessive-Compulsive Drinking Scale (OCDS) once a week for 5 weeks (the week before treatment, during the second, third and fourth treatment weeks, and the week after treatment). A, anterior; P, posterior; R, right; L, left; a, anode; c, cathode; BS, brain stimulation.

The cathode was placed above F3 (according to the EEG international 10–20 system), corresponding to the left dlPFC, and the anode was positioned above the right dlPFC (F4). For the sham stimulation procedure, the stimulator automatically switched off after 30 s of either anodal or cathodal stimulation yielding sensations typically elicited by tDCS. Sham- or real tDCS was applied once a day, every other day, including weekends, until completion of 10 sessions (Figure 1).

### Craving assessment: 5-items OCDS

Craving was scored with a brief scale composed of 5 items (1, 2, 4, 5, and 13) from the Obsessive-Compulsive Drinking Scale (OCDS) (Anton et al., 1995, 1996; Anton, 2000), which assesses craving in a narrow sense according to de Wildt et al. (2005).

Questions of this brief scale allow quantification of thoughts and feelings (obsessions), and behavioral intentions (de Wildt et al., 2005), and are answered on a scale ranging from 0 to 4, resulting in a total score between 0 and 20. They ask how much of a person's time (total per day), when the drug is not used, is occupied by thoughts, ideas, desires, or impulses related to alcohol and its effects; how frequently these thoughts, ideas, desires, or impulses related to alcohol and its effects occur; how much distress or disturbance these ideas, thoughts, impulses or desire related to alcohol use cause when the person is under withdrawal; how much effort the person has to make to resist these thoughts, ideas, desires, or impulses, or how much energy he/she has to spend to think of something else when they enter the mind under withdrawal; and finally ask about the person's drive to use alcohol.

This scale was applied in the week before the beginning of the real or sham-tDCS treatment (1st measurement), during the treatment over ~3-weeks (2nd, 3rd, and 4th measurements) and in the week after the end of the brain stimulation protocol (5th measurement).

### 3-Months follow-up: alcohol use relapses

After their discharge from the hospital, patients from sham- and real tDCS groups were followed-up for 3 months, corresponding to a period of initial remission according to DSM-5, regarding alcohol use relapses. Alcohol use relapse here was considered as the first episode of return to the previous uncontrolled pattern of alcohol use (drinks per day) (Klauss et al., 2014).

### Procedures

**Figure 2:**
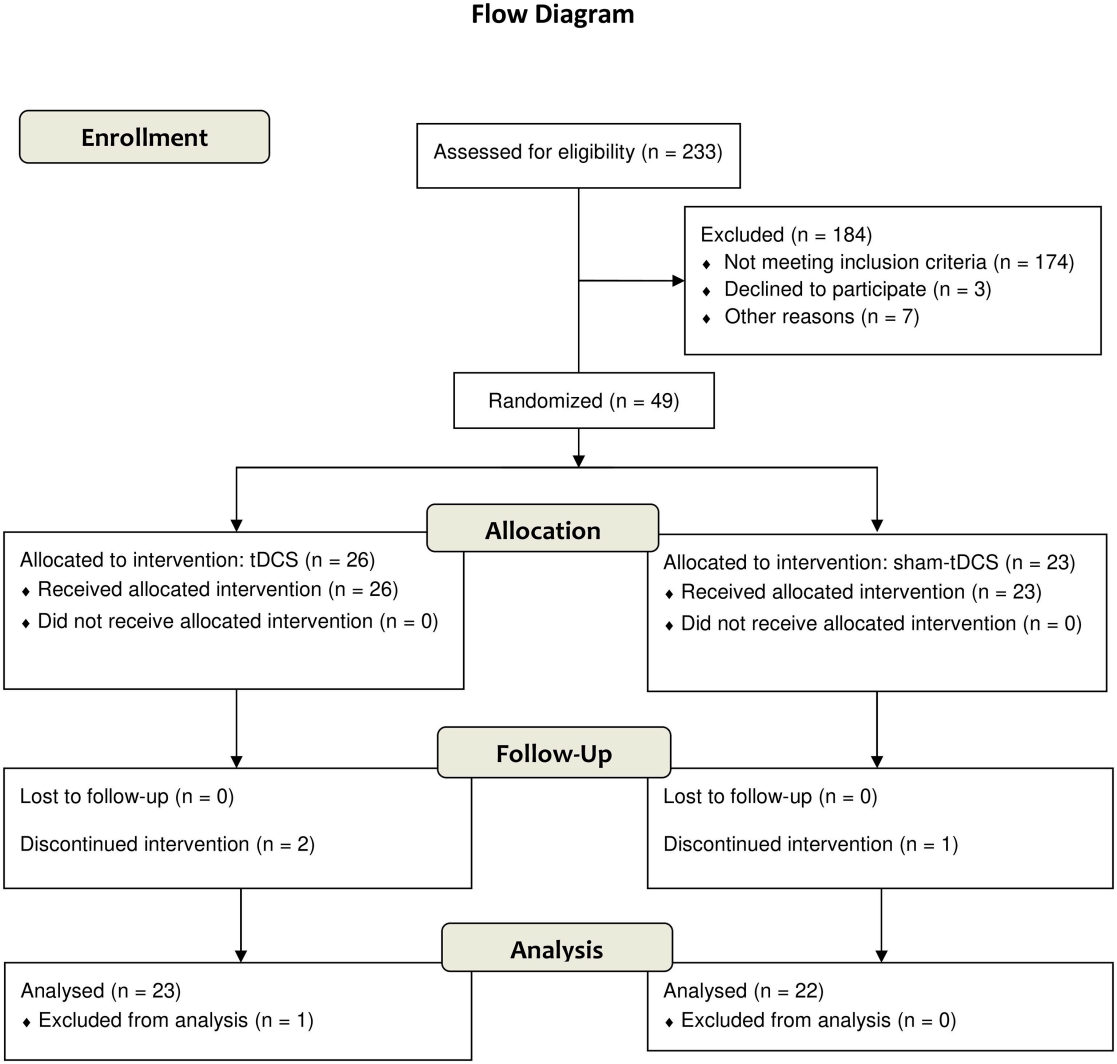
Flow diagram according to CONSORT 2010.

Those patients who were eligible (Figure 2) for study participation according to the inclusion and exclusion criteria described above and agreed to participate in this study signed an informed consent form (Figure 1). All data were originally acquired from participants participating in a randomized sham-controlled double-blind clinical trial to investigate the efficacy of tDCS treatment of alcohol dependence.

After global physical and clinical examination, subjects were randomly assigned to one of the two intervention groups (sham- and real tDCS) in a 1:1 ratio using a computer-generated block randomization sequence that was kept with the unblinded study coordinator (not involved in recruitment) and only revealed to the co-investigator conducting treatments immediately before the first session.

Craving (5-items OCDS) was measured before and after completion of the treatment and once per week during the 3-weeks treatment, resulting in five measurements (Figure 1). Alcohol use relapses after hospital discharge were verbally obtained from patients, families or caregivers.

Participants and experimenters were blinded for brain stimulation assignments from the beginning of the study protocol up to the end of the 3-months follow-up after the end of sham-tDCS or real tDCS treatment, configuring a double-blind experimental design.

### Statistical analysis

We powered the study for a small effect size given our hypothesis that tDCS would be associated with a relevant reduction in craving scores. Thus, assuming a small effect size of 0.3 specified for SPSS in G^*^Power 3.1 for a repeated measure (5 measures) within-between interaction analysis of variance (ANOVA) as principal statistical test for the craving analysis with a power of 80%, a two-sided probability of a type I error of 5%, a minimum of 38 subjects would be necessary; however to account for waiving or dropouts expected to be very common in this condition, we increased the estimated sample to ~20%, resulting in 45 to 46 subjects (22 to 23 subjects in each group).

Age, patterns of alcohol use and 5-items OCDS were normally distributed according to D'Agostino & Pearson normality test, thus they were analyzed by parametric tests.

Besides the two-way ANOVA (sham-tDCS and tDCS groups as between-subjects factor) with repeated measures (five time-points as within-subjects factor) followed by Bonferroni's multiple comparisons as *post-hoc* test, linear regression analyses were done over craving scores obtained over the respective five time-points for both groups and the slopes of the respective curves were compared using a modified version of the *t*-test according to Zar (1984), which is equivalent to analysis of covariance. Additional comparisons between initial and final OCDS scores were done by paired *t*-tests for each group, and differences between final and initial scores were compared between sham-tDCS and tDCS groups with unpaired *t*-tests. Effect sizes were calculated using Cohen's d and corrected by Hedges's *g*_*s*_ for unpaired and Hedges' g_av_ for paired *t*-tests (Lakens, 2013).

Age and patterns of alcohol use were compared between groups by unpaired *t*-test. For all other non-parametric data, Chi-square or Fisher tests were used to compare results between sham and real tDCS groups.

A two-tailed *p*-value of 0.05 or less was considered to indicate statistical significance. SPSS Statistics Base 24.0 (SPSS Inc., USA) and GraphPad Prism 7.0 (GraphPad Software Inc, USA) were employed for statistical analysis and graphic presentations.

## Results

### Baseline data

Baseline socio-demographic characteristics and patterns of drug use are presented in Tables 1, 2.

**Table 1:** Socio-demographic characteristics of the total sample of alcoholics (*n* = 45) and subdivided in subjects submitted to bilateral repetitive transcranial Direct Current Stimulation (tDCS: cathode left/anode right dorsolateral Prefrontal Cortex, 2 mA, 35 cm^2^, 20 min, 10 sessions, every other day, *n* = 23) or placebo (sham-tDCS: *n* = 22).

	**Alcoholics (*****n*** = **45)**		**Groups**			***p*-value**
			**Sham-tDCS (*n* = 22)**	**tDCS (*n* = 23)**		
Age [mean (*SD*)]		44.9 (11.1)	43.5 (10.2)	46.3 (12.0)	*t*_(43)_ *=* −0.84	0.40
Gender *n* (%)	Male Female	37 (82.2%) 8 (17.8%)	19 (86.4%) 3 (13.6%)	18 (78.3%) 5 (21.7 %)	Fisher = 0.7	0.38
Years of education *n* (%)	Up to 5 Between 6 and 9 Between 10 and 13 Above 13 Not reported	22 (48.9%) (19M:3F) 5 (11.1%) (5M:0F) 10 (22.2%) (7M:3F) 7 (15.6%) (5M:2F) 1 (2.2%) (1M:0F)	9 (40.9%) (8M:1F) 4 (18.2%) (4M:0F) 5 (22.7%) (4M:1F) 4 (18.2%) (3M:1F) 0 (0.0%) (0M:0F)	13 (56.5%) (11M:2F) 1 (4.3%) (1M:0F) 5 (21.7%) (3M:2F) 3 (13.0%) (2M:1F) 1 (4.3%) (1M:0F)	*X*_2_ *T* = 3.65 X_2_ *M* = 3.59 X_2_ *F* = 0.18	0.46 0.46 0.92
Employment situation *n* (%)	Formal job Informal job Unemployed Freelance Retired Disease benefit Not reported	8 (17.8%) (8M:0F) 2 (4.4%) (2M:0F) 21 (46.7%) (16M:5F) 4 (8.9%) (3M:1F) 4 (8.9%) (4M:0F) 3 (6.7%) (3M:0F) 3 (6.7%) (1M:2F)	6 (27.3%) (6M:0F) 0 (0.0%) (0M:0F) 10 (45.5%) (8M:2F) 2 (9.1%) (2M:0F) 2 (9.1%) (2M:0F) 0 (0.0%) (0M:0F) 2 (9.1%) (1M:1F)	2 (8.7%) (2M:0F) 2 (8.7%) (2M:0F) 11 (47.8%) (8M:3F) 2 (8.7%) (1M:1F) 2 (8.7%) (2M:0F) 3 (13.0%) (3M:0F) 1 (4.3%) (0M:1F)	*X*_2_ *T* = 7.36 *X*_2_ *M* = 8.31 *X*_2_ *F* = 0.75	0.29 0.22 0.69
Marital state *n* (%)	Single Married or Common-law Divorced Widow Not reported	22 (48.9%) (19M:3F) 14 (31.1%) (11M:3F) 6 (13.3%) (6M:0F) 2 (4.4%) (1M:1F) 1 (2.2%) (0M:1F)	7 (31.8%) (6M:1F) 7 (31.8%) (6M:1F) 6 (27.3%) (6M:0F) 1 (4.5%) (1M:0F) 1 (4.5%) (0M:1F)	15 (65.2%) (13M:2F) 7 (30.4%) (5M:2F) 0 (0.0%) (0M:0F) 1 (4.3%) (0M:1F) 0 (0.0%) (0M:0F)	*X*_2_ *T* = 9.89 *X*_2_ *M* = 9.65 *X*_2_ *F* = 2.31	0.04^*^ 0.02^*^ 0.51
Race *n (%)*	White Brown Black	23 (51.5%) (18M:5F) 14 (31.1%) (13M:1F) 8 (17.8%) (6M:2F)	12 (54.5%) (10M:2F) 6 (27.3%) (6M:0F) 4 (18.2%) (3M:1F)	11 (47.8%) (8M:3F) 8 (34.8%) (7M:1F) 4 (17.4%) (3M:1F)	*X*_2_ *T* = 0.31 *X*_2_ *M* = 0.27 *X*_2_ *F* = 0.75	0.86 0.87 0.69
Tobacco use *n (%)*	Yes No	23 (51.1%) (18M:5F) 22 (48.9%) (19M:3F)	11 (50.0%) (10M:1F) 11 (50.0%) (9M:2F)	12 (52.2%) (8M:4F) 11 (47.8%) (10M:1F)	Fisher *T* = 1.0 Fisher *M* = 0.75 Fisher *F* = 0.46	0.56 0.43 0.29
Used illicit drugs^#^ *n (%)*	Yes No	6 (13.3%) (4M:2F) 39 (86.7%) (33M:6F)	2 (9.1%) (2M:0F) 20 (90.9%) (17M:3F)	4 (17.4%) (2M:2F) 19 (82.6%) (16M:3F)	Fisher *T* = 0.67 Fisher *M* = 1.0 Fisher *F* = 0.46	0.35 0.68 0.36

* *p = 0.05 when compared to sham*.

# *she/he had experienced marijuana in the adolescence. T, total; M, male; F, female*.

**Table 2:** Patterns of alcohol use, impression of what treatment they were in and confidence of this impression, and adverse events, for the total sample of alcoholics (*n* = 45) and subdivided in subjects submitted to bilateral repetitive transcranial Direct Current Stimulation (tDCS: cathode left/anode right dorsolateral Prefrontal Cortex, 2 mA, 35 cm^2^, 20 min, 10 sessions, every other day, *n* = 23) or placebo (sham-tDCS: *n* = 22).

		**Alcoholics (*n* = 45)**	**Groups**			***p*-value**
			**Sham-tDCS (*n* = 22)**	**tDCS (*n* = 23)**		
**ALCOHOL USE**						
Age at onset of alcohol use [mean (*SD*)]		16.2 (5.7)	16.9 (6.0)	15.5 (5.4)	*t*_(43)_ = −0.56	0.40
Amount of alcohol used (drinks/day) [mean (*SD*)]		17.9 (14.3)	15.5 (15.0)	20.3 (13.4)	*t*_(43)_ = −1.13	0.26
Days of abstinence before study [mean (*SD*)]		33.0 (12.4)	32.9 (12.5)	33.0 (12.7)	*t*_(43)_ = −0.02	0.98
**IMPRESSION** ***n (%)***						
Sham (placebo)		3 (6.7%)	2 (9.1%)	1 (4.3%)	Fisher = 0.61	0.48
tDCS treatment		42 (93.3%)	20 (90.9%)	22 (95.7%)		
Confidence in their impression *n* (%)	(1) None	0 (0%)	0 (0%)	0 (0%)	*X*_2_ = 5.11	0.16
	(2) Little	3 (6.7%)	1 (4.5%)	2 (8.7%)		
	(3) Medium	4 (8.9%)	3 (13.6%)	1 (4.3%)		
	(4) Very confident	23 (51.1%)	8 (36.4%)	15 (65.2%)		
	(5) Extremely confident	15 (33.3%)	10 (45.5%)	5 (21.7%)		
**ADVERSE EVENTS^#^***n (%)*****						
None		16 (35.6%)	9 (40.9%)	7 (30.4%)	Fisher = 0.54	0.34
Tingling in the scalp		29 (64.4%)	13 (59.1%)	16 (69.6%)		

# *No other adverse event asked was registered (headache, neck and scalp pain, itching, skin redness, burning sensation of the scalp, sleepiness, acute mood changes, trouble concentrating)*.

AUD subjects were middle aged, with an average of 44.9 years old in the total sample, mostly with low schooling scores (48.9% of them had up to 5 years of education), unemployed (46.7%), single (48.9%) and of white skin color (51.5%) (Table 1). In addition, more than half of them (51.5%) were tobacco smokers and the majority (86.7%) had no experience with other drugs besides alcohol (Table 1).

Except for the marital state, which showed differences between groups (*p* = 0.04), mostly because of the greater proportion of single subjects in the tDCS group and of divorced subjects in the sham-tDCS group, no other socio-demographic parameter differed between groups (Table 1).

They started to use alcohol on average at 16.2 years of age, consumed on average 17.9 drinks per day, and they were about 33 days abstinent before the beginning of the experimental protocol (Table 2). None of these characteristics differed between sham and real tDCS groups (Table 2).

Patients were kept in a restrictive environment for drug use during the treatment. They were blinded for tDCS treatment. When they were asked about their impression of what treatment they had received at the end of the treatment, 42 (93.3%) subjects answered they were exposed to real tDCS (Table 2). That is, only 3 (6.7%) subjects answered they received sham-tDCS. Two of them were from the sham-tDCS group and one from real tDCS group. From the sham-tDCS group, 20 out of 22 (90.9%) answered they were receiving real tDCS treatment, and almost all subjects (95.7%) from the real tDCS group answered positively. When they were asked how confident they were regarding treatment condition, in the total sample 38 (84.4%) were very to extremely confident, 18 (81.9%) from sham-tDCS group and 20 (87.9%) from tDCS group. There were no statistically significant differences between groups for both parameters, impression and confidence (Table 2).

### Adverse events

We asked subjects about the following adverse effects: headache, neck and scalp pain, tingling, itching, skin redness, burning sensation of the scalp, sleepiness, acute mood changes, trouble concentrating, and others (Brunoni et al., 2011) after treatment. From these potential events, a tingling sensation was reported by 29 subjects (64.4%) in the total sample, and quite equally by sham-(13 subjects, 59.1%) and real tDCS (16 subjects, 69.6%) groups (Table 2). Nine subjects (40.9%) from the sham-tDCS group and seven from the real tDCS group (30.4%) reported no events at all. No other adverse events were reported by AUD patients from both groups in this study and no significant difference was found between groups (Table 2).

### Craving: 5-items OCDs

**Figure 3:**
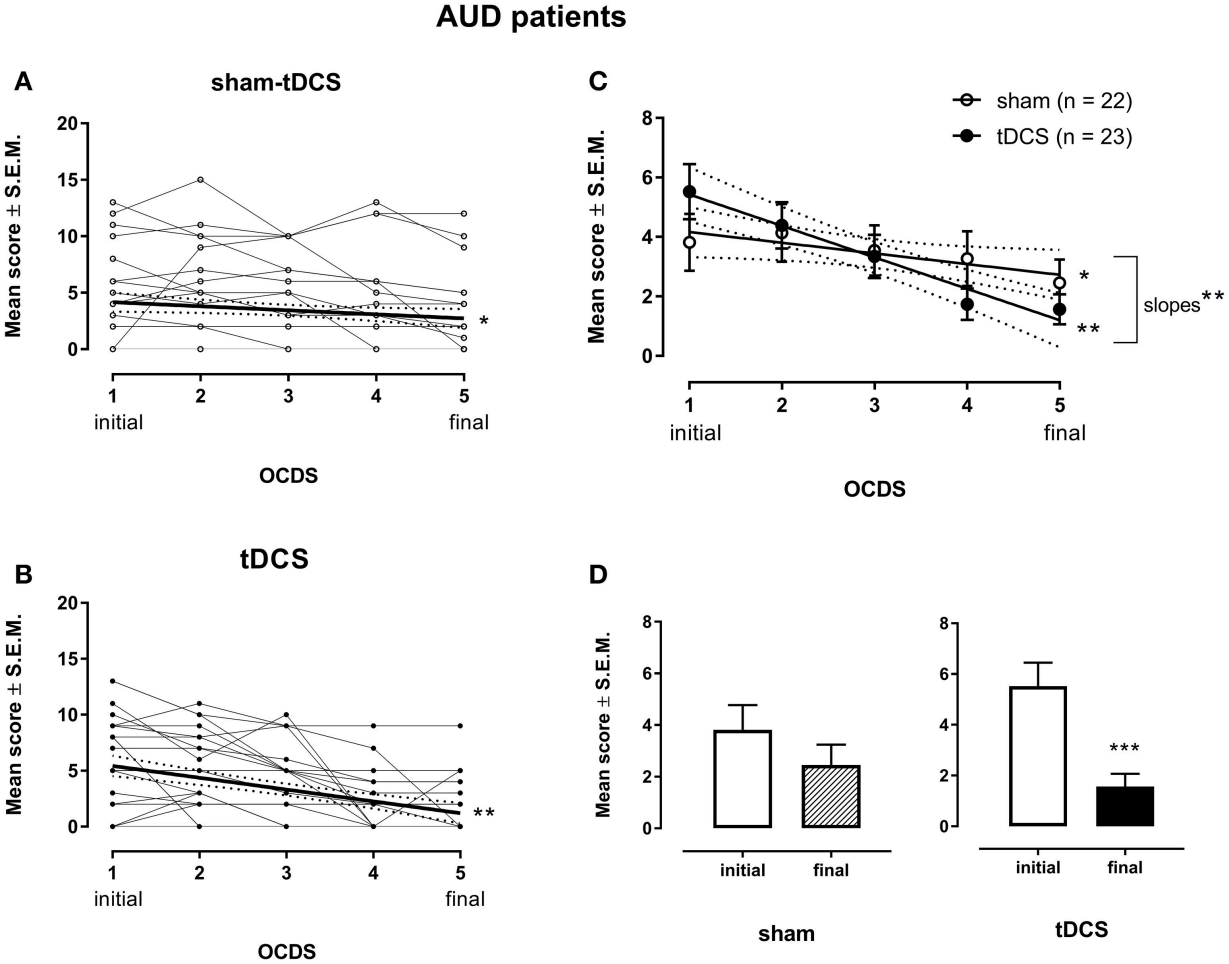
Craving is shown as the 5-items from the Obsessive-Compulsive Drinking Scale (OCDS) scores in the week before treatment (1 initial), the second (2), third (3) and fourth (4) weeks during the treatment, and the week after treatment (5 final) with bilateral repetitive transcranial Direct Current Stimulation (tDCS, 2 mA, 35 cm^2^: cathode left/anode right over the dorsolateral Prefrontal Cortex; stimulation for 20 min every other day in a total of 10 sessions; *n* = 23) or placebo (sham-tDCS; *n* = 22) in Alcohol Use Disorder (AUD) patients individually (**A** for sham-tDCS; **B** for tDCS subjects) and by their mean scores ± standard error of means (S.E.M.) **(C)**. Linear regression for sham-tDCS: ^*^p < 0.05; Linear regression for the real tDCS group: ^**^p < 0.005, slope difference: ^**^p < 0.005. Mean scores of craving depicted in the week before and the week after treatment in the real and sham-tDCS groups are shown in **(D)**. ^***^p = 0.0005 when compared to baseline craving in the real tDCS group (paired *t*-test).

A two-way ANOVA with repeated measures was conducted to examine the intervention effect on craving (Figure 3). Both tDCS and sham-tDCS groups differed in OCDS scores over time (Figures 3A–C). The ANOVA shows a significant five time-points within-subject effect [*F*_(4, 172)_ = 13.15, *p* < 0.0001, η^2^_*p*_ = 0.23, $\eta_{\text{G}}^{2}$ = 0.068] and a significant interaction between groups and five time-points OCDS measurements [*F*_(4, 172)_ = 3.91, *p* = 0.005, $\eta_{\text{p}}^{2}$ = 0.08, $\eta_{\text{G}}^{2}=$ 0.021], suggesting that craving scores changed differently between groups during the intervention. Bonferroni's multiple comparisons tests showed that OCDS scores were significantly smaller in the 3rd, 4 and 5th measurements when compared to the 1st measurement (adjusted *p*-value < 0.01, 0.0001 and 0.0001, respectively) and in the 4th and 5th measurements when compared to the 2nd measurement (adjusted *p*-value < 0.001 and 0.0001, respectively) in the real tDCS group only.

Thus, craving scores decreased linearly from baseline (week before treatment) to the week after treatment in both groups [Linear regression for the sham-tDCS group: *Y* = 4.523–0.3591X, *r*^2^ = 0.7872, *F*_(1, 3)_ = 11.1, *p* = 0.0447; Linear regression for the real tDCS group: *Y* = 6.483–1.057X, *r*^2^ = 0.9644, *F*_(1, 3)_ = 81.3, *p* = 0.0029] (Figure 3C). The difference between the slopes from both groups was statistically significant [*F*_(1, 6)_ = 19.19, *p* = 0.00047], showing that the decrease of OCDS scores was significantly larger in the tDCS group (Figure 3C).

When comparing craving scores obtained before (initial) and after (final) treatment by paired *t*-tests, a statistically significant difference {*t*_(22)_ = 4.06, *p* = 0.0005, 95% CI [1.93, 5.98]} was observed for the real tDCS group only (Figure 3D), showing that OCDS scores were significantly smaller than baseline values after 10 sessions of bilateral cortical DC stimulation. The corrected effect size for the paired *t*-tests between initial and final craving scores of the tDCS group by Hedges's *g*_*av*_was 1.07 (initial mean score = 5.52, *SD* = 4.44; final mean score = 1.56, *SD* = 2.41).The effect size calculated indicates that after controlling for individual differences, the likelihood that OCDS scores of an AUD patient under tDCS treatment are lower for the final than for initial mean score is 80%.

The corrected effect size by Hedges's *g*_*av*_ for the sham-tDCS group was 0.32 {initial mean = 3.82, *SD* = 4.47; final mean = 2.46, *SD* = 3.67; *t*_(21)_ = 1.54, *p* = 0.14; 95% CI [−0.48, 3.20]}. The effect size calculated indicates that after controlling for individual differences, the likelihood that scores of an AUD patient under sham-tDCS treatment are lower for the final than for initial mean craving score is 63%.

Considering the effects size between initial and final craving scores of 1.07 of real tDCS group and 0.32 of sham-tDCS group, the effect size was ~3.33-fold larger in the real tDCS group over sham-tDCS group.

When comparing the mean change scores contrasting data obtained after 10 sessions (final) vs. baseline (initial) between groups (sham-tDCS vs. tDCS), the respective unpaired *t*-test resulted in *p* = 0.056 {*t*_(43)_ = 1.96, 95% CI [0.52, 1.48]}. The corrected effect size by Hedges's *g*_*s*_ for two independent samples was 0.58 (mean sham-tDCS difference = −1.36, *SD* = 4.15; mean tDCS difference = −3.96, *SD* = 4.68). The effect size indicates that the chance for a randomly selected pair of subjects, the probability of a lower score of an AUD patient from tDCS-group, as compared to the score of an AUD patient from the sham-tDCS group, is 66%.

### Alcohol use relapses

Five AUD patients were lost to the follow-up, all from the sham-tDCS group, most of them because they and/or their relatives could not be reached after many attempts. At least two of them are homeless.

**Figure 4:**
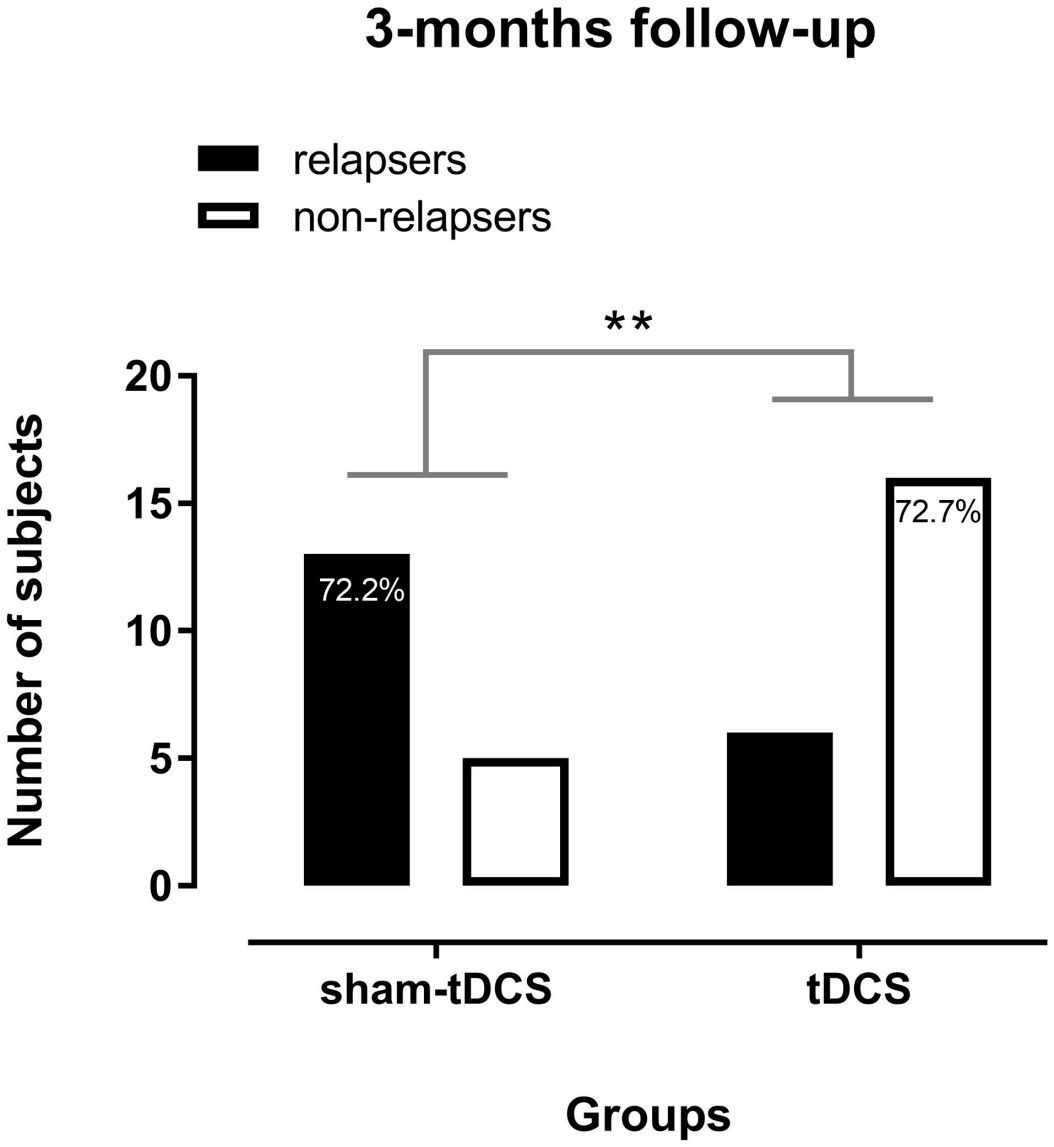
Alcohol use relapses in alcohol use disorder (AUD) patients in the 3 months follow-up after 10 sessions of sham- (*n* = 18) or real tDCS (*n* = 22) applied over the bilateral dorsolateral Prefrontal Cortex. ^**^p = 0.01 (Fisher's exact test).

Alcohol use relapses up to 3 months after the end of 10 sessions of brain stimulation were significantly higher (*p* = 0.01, Fisher's exact test) in the sham-tDCS group and, by contrast, alcohol abstinence was proportionately larger in the real tDCS group. 72.2% of patients from the sham-tDCS group relapsed and 72.7% of patients from the real tDCS group were abstinent at the end of the 3-months follow-up (Figure 4). From those who relapsed, 68.4% were from the sham-tDCS group and from those who kept abstinence 76.2% were from the real tDCS group.

## Discussion

Regarding demographic data, except for the marital state, AUD patients from sham- and real-tDCS groups were matched by socio-demographic characteristics such as age, gender, schooling and employment conditions and by characteristics of alcohol use, especially the age at the onset of alcohol use and days of abstinence before tDCS. Therefore, the craving score reduction in AUD patients treated with 10 sessions of bilateral prefrontal tDCS was obtained in comparison to a well-matched placebo (sham) control group. Craving scores progressively decreased in both groups over the course of sham-tDCS or real tDCS treatment, but they were significantly reduced only in the real tDCS group at the end of the 10-sessions applications.

Alcohol dependency is a complex disease, involving several brain areas (Preti et al., 2014) and a variety of chemical changes (Koob and Volkow, 2016). This makes therapeutic strategies for its treatment complex, and usually of limit success. In this context, adjunctive therapies such as brain stimulation may play a notable role (Higgins and George, 2009).

Brain regions involved in the rewarding system (Wilson et al., 2004; Kalivas and Volkow, 2005; Volkow et al., 2011), such as the nucleus accumbens, the amygdala, the anterior cingulate, the orbitofrontal cortex, and the dlPFC, are involved in drug addiction (Vetulani, 2001; Gardner, 2011; Koob and Volkow, 2016). Thus, strategies focused on these brain regions may help to change rewarding characteristics of drug use. Indeed, DC stimulation targeting the dlPFC has shown promising applicability as adjunctive approach in the treatment of alcohol dependence, as it reduces relapse and craving (Jansen et al., 2013; Klauss et al., 2014; Batista et al., 2015). These effects seem not to be restricted to alcohol dependency, but are also accomplished in crack-cocaine dependence, and other addictive conditions (Boggio et al., 2008; Fregni et al., 2008; Goldman et al., 2011).

Most clear effects of cortical DC stimulation have been observed when tDCS was applied consecutively in multiple sessions. Four sessions of unilateral anodal tDCS over the dlPFC combined with cognitive bias modification tended to decrease relapses to the alcohol use after 1 year (den Uyl et al., 2016). So far, in our studies on drug addiction we have applied five sessions of tDCS on consecutive days or every other day over the dlPFC (Klauss et al., 2014; Batista et al., 2015).

In our previous tDCS study in AUD patients, in which only 5 stimulation sessions were conducted, out of 33 outpatients, 17 were randomly distributed to the sham-tDCS group and 16 to the active tDCS group. Of these, 15 patients in the sham-tDCS group and eight in the active tDCS group relapsed to alcohol use during treatment or in the 6-month follow-up (Klauss et al., 2014). In this study, no between group differences were found for craving changes. However, in that outpatient study tDCS was applied for only five consecutive days (over a short period of time), and craving was scored before and after 1 week of treatment only due to logistic reasons. Therefore, although significant result was found on relapse, 5 tDCS sessions may have been too short to change craving response in AUD patients because other studies have shown better results with longer brain stimulation sessions (Valle et al., 2009; Kuo et al., 2014).

In the present study, in AUD patients admitted to specialized clinics, tDCS (or sham-tDCS) was applied every other day in a total of 10 applications, which was supposed to be a more efficient protocol, and allowed to monitor craving more accurately during the course of the treatment.

Craving scores were slightly decreased in the sham-tDCS group, showing that regular biopsychosocial and behavioral treatment conducted in the clinic was efficient. However, the reduction of craving was relevantly larger in AUD patients receiving real tDCS treatment, as it was shown by the respective statistical analyses.

To better understand the magnitude of this effect with regard to its clinical relevance, we calculated corrected effect sizes according to Hedges's *g*_*s*_ (Lakens, 2013). According to Lakens (2013), the effect size of 0.3 (small to medium effect size according to Cohen's convention) (Cohen, 1992) obtained in the sham-tDCS group means that craving scores of 63% of the AUD patients under regular treatment for alcohol dependence will be below the mean score observed before treatment. The resulting number needed to treat for the sham tDCS group is 10.6, corresponding to a favorable outcome in approximately 9.4% of the patients when compared to baseline.

For real tDCS, an effect size of 1.1 (large effect size according to Cohen's convention) (Cohen, 1992) was achieved, which means that 80% of AUD patients under regular treatment for alcohol dependence added by repetitive DC stimulation showed craving scores below the mean score observed before treatment. The resulting number needed to treat for the real tDCS group is 3.5 patients, referring to a favorable outcome in 28.6% of all patients compared to baseline.

These analyses showed that tDCS was more favorable than regular treatment (placebo) in about 3-fold in reduction of craving to the alcohol use, which was complemented by between-groups comparison on mean score changes finding a medium effect size in favor of tDCS treatment (Cohen, 1992). These evidence of favorable outcome of repetitive tDCS on craving behavior hopefully will be of clinical help in the treatment of alcohol dependence.

Furthermore, most patients (72.7%) from the real tDCS group were able to maintain alcohol abstinence over 3 months after treatment, thus being in early remission of AUD according to DSM-5, whereas fewer patients from the sham-tDCS group (27.8%) were abstinent after this period. In fact, most sham-tDCS patients relapsed in this period. In our previous study (Klauss et al., 2014), about 56.25% of AUD patients were abstinent over ~3 months after 5 sessions of the same tDCS protocol, suggesting that 10 sessions of brain stimulation are more effective to sustain alcohol abstinence.

There are limitations of this study that must be considered. Although our sample sizes were sufficient for our designed statistical analysis, they are still small and restricted by inclusion and exclusion criteria, which limit generalizability. A huge sample (233 subjects) of drug users admitted in three clinics for drug dependence treatment was interviewed but only 49 (21%) AUD patients were eligible for this study according to our criteria (Figure 2). Future investigations should extend to a more unrestricted AUD population to increase the generalizability of the potential clinical application of DC stimulation in the treatment of alcohol dependence. It must be mentioned that surrogate analysis of cognitive performance, clinical outcomes such as anxiety and depression symptoms, quality of life, and electrophysiological data that have been collected needs to be processed to help to understand the extension of neuromodulatory effects of the tDCS and the mechanisms that may underlie these effects. They were not included here because they constitute an extensive volume of data that would go beyond the objective of this report. And, finally, we must underscore that this study shows effects of one specific montage of tDCS application and explored its extended number of sessions. Effects of other electrode montages and sizes, and different parameters of brain stimulation need to be explored in future studies.

In summary, this study shows that 10 sessions of bilateral tDCS over the dlPFC (cathodal right, and anodal left) decreased craving and relapses for alcohol use in severe AUD patients in more efficiently degree than regular treatment for alcohol dependence alone. Therefore, this stimulation protocol is a promising, non-expensive, add-on clinical tool, which could help to reduce alcohol craving, and consequently facilitate alcohol use cessation in severe AUD patients.

## Author contributions

We hereby submit an original research article entitled, A randomized placebo-controlled trial of tDCS as an add-on treatment of alcohol use disorder for consideration by Frontiers in Pharmacology, section Neuropharmacology. This study provides evidence that ten sessions of bilateral tDCS over the dorsolateral Prefrontal Cortex (cathodal right and anodal left) decreased craving and relapses for alcohol use in severe AUD patients in more efficiently degree than regular treatment for alcohol dependence alone. We confirm that this work is original and has not been published elsewhere, nor is it currently under consideration for publication elsewhere. All authors (JK, QSA, LVF, MAN, EMN-P) have read and approved the manuscript for submission; have made a substantial contribution to the conception, design, gathering, analysis and/or interpretation of data and a contribution to the writing and intellectual content of the article; and acknowledge that they have exercised due care in ensuring the integrity of the work. None of the original material contained in the manuscript has been submitted for consideration nor will any of it be published elsewhere except in abstract form in connection with scientific meetings.

### Conflict of interest statement

MN is member of the scientific advisory board of Neuroelectrics. The remaining authors declare that the research was conducted in the absence of any commercial or financial relationships that could be construed as a potential conflict of interest.
